# Supplementary material for: Intact protein barcoding enables one-shot identification of CRISPRi strains and their metabolic state
Source: Cell Rep Methods. 2024 Nov 26;4(12):100908. doi: 10.1016/j.crmeth.2024.100908 (PMC11704613; doi:10.1016/j.crmeth.2024.100908)
Supplement: Document S1. Figures S1 and S2 [file mmc1.pdf]

Cell Reports Methods, Volume *4*

Supplemental information

Intact protein barcoding enables one-shot identification of CRISPRi strains and their metabolic state

Vanessa Pahl, Paul Lubrano, Felicia Troßmann, Daniel Petras, and Hannes Link

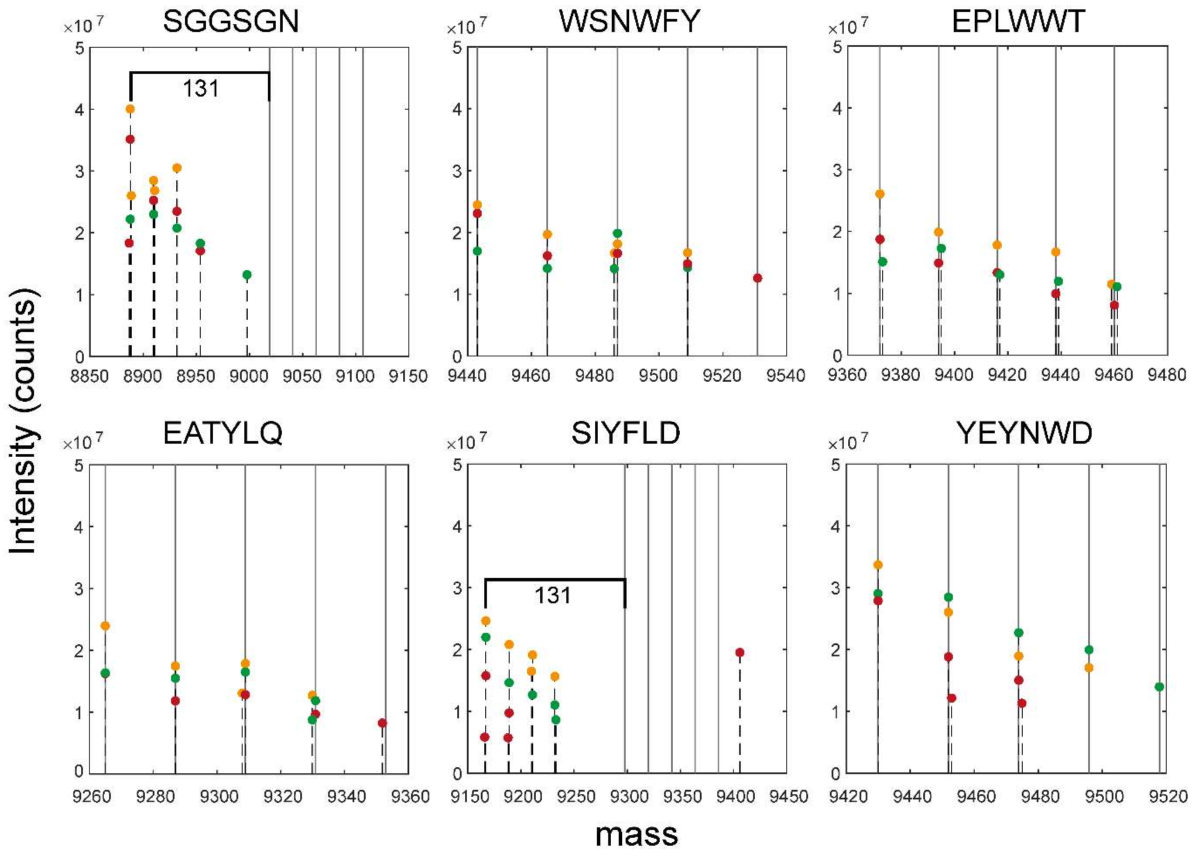

Figure S1. Monoisotopic masses of six ubiquitin barcodes determined by spectral deconvolution of FI-MS data, Related to Figure 3. Shown are the 5 monoisotopic masses with highest summed intensity from n = 3 samples. Dots with different colour and dotted lines are deconvoluted masses of n = 3 replicates. Solid lines are theoretical masses of ubiquitin with the N-terminal barcodes shown above the graph, with 0-4 sodium adducts.

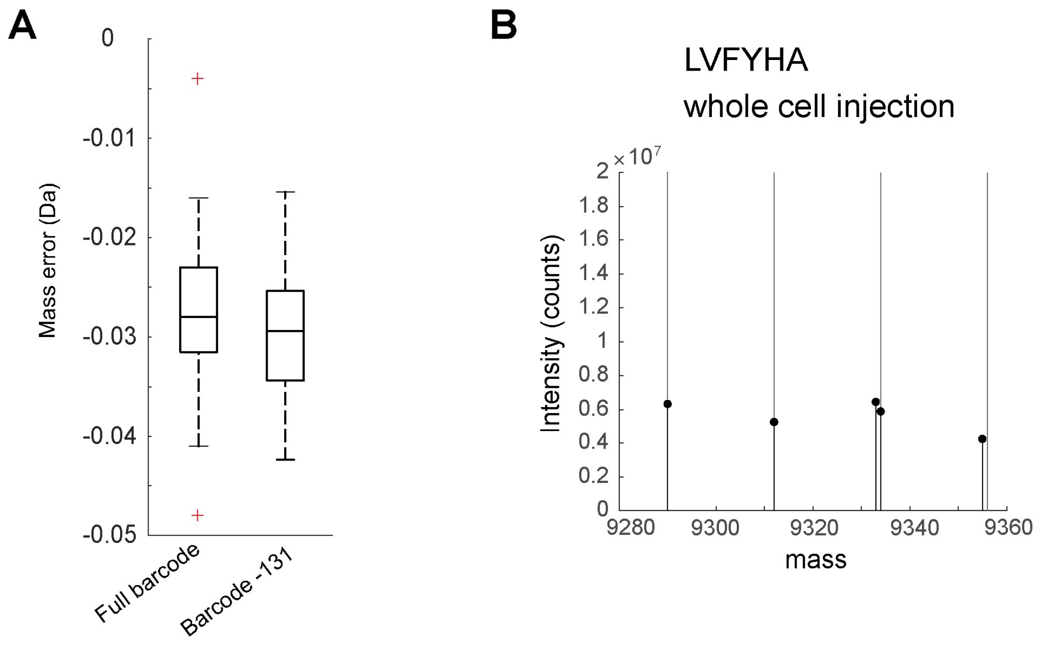

Figure S2. Mass accuracy of protein barcode detection and analysis of the ubiquitin-LVFYHA barcode in whole living cells, Related to Figure 4. A) Mass accuracy of protein barcode detection after spectral deconvolution. Shown is the error for 48 barcodes with correct monoisotopic mass (full barcode), and 38 barcodes with correct monoisotopic mass -131 due to methionine cleavage (barcode -131). **B)** The CRISPRi control strain expressing LVFHA-ubiquitin was diluted in 1:8 M9-medium: water and 3 μL were directly injected into the mass spectrometer. Cells were heated to 90°C in the heat exchanger of the column oven. Shown are 5 deconvoluted masses with highest summed intensities. Dots with dotted lines are deconvoluted masses. Solid lines are theoretical masses of LVFYHA-ubiquitin, with 0-4 sodium adducts.
