# Supplementary material for: Intact protein barcoding enables one-shot identification of CRISPRi strains and their metabolic state
Source: Cell Rep Methods. 2024 Nov 26;4(12):100908. doi: 10.1016/j.crmeth.2024.100908 (PMC11704613; doi:10.1016/j.crmeth.2024.100908)
Supplement: Data S1. Mass spectra of cell extracts from E. coli strains expressing ubiquitin barcodes, related to Figures 1 and 3 [file mmc7.pdf]

Data S1 - **Mass spectra of cell extracts from *E. coli* strains expressing ubiquitin barcodes, Related to Figure 1 and Figure 3.** Shown are the MS1 spectra in positive mode from 0 to 1700 mass-to-charge ratio (m/z) measured with FI-MS. Black spectra show the relevant strain and orange spectra are a reference.

Page 2: Full MS1 spectrum of the LVFYHA-ubiquitin strain (black), and the control strain (orange).

Page 3: Full MS1 spectrum of the DapE strain (black), and the LVFYHA-ubiquitin strain (orange).

Page 4: Full MS1 spectrum of the Dxr strain (black), and the LVFYHA-ubiquitin strain (orange).

Page 5: Full MS1 spectrum of the HisD strain (black), and the LVFYHA-ubiquitin strain (orange).

Page 6: Full MS1 spectrum of the Icd strain (black), and the LVFYHA-ubiquitin strain (orange).

Page 7: Full MS1 spectrum of the LeuB strain (black), and the LVFYHA-ubiquitin strain (orange).

Page 8: Full MS1 spectrum of the ThrC strain (black), and the LVFYHA-ubiquitin strain (orange).

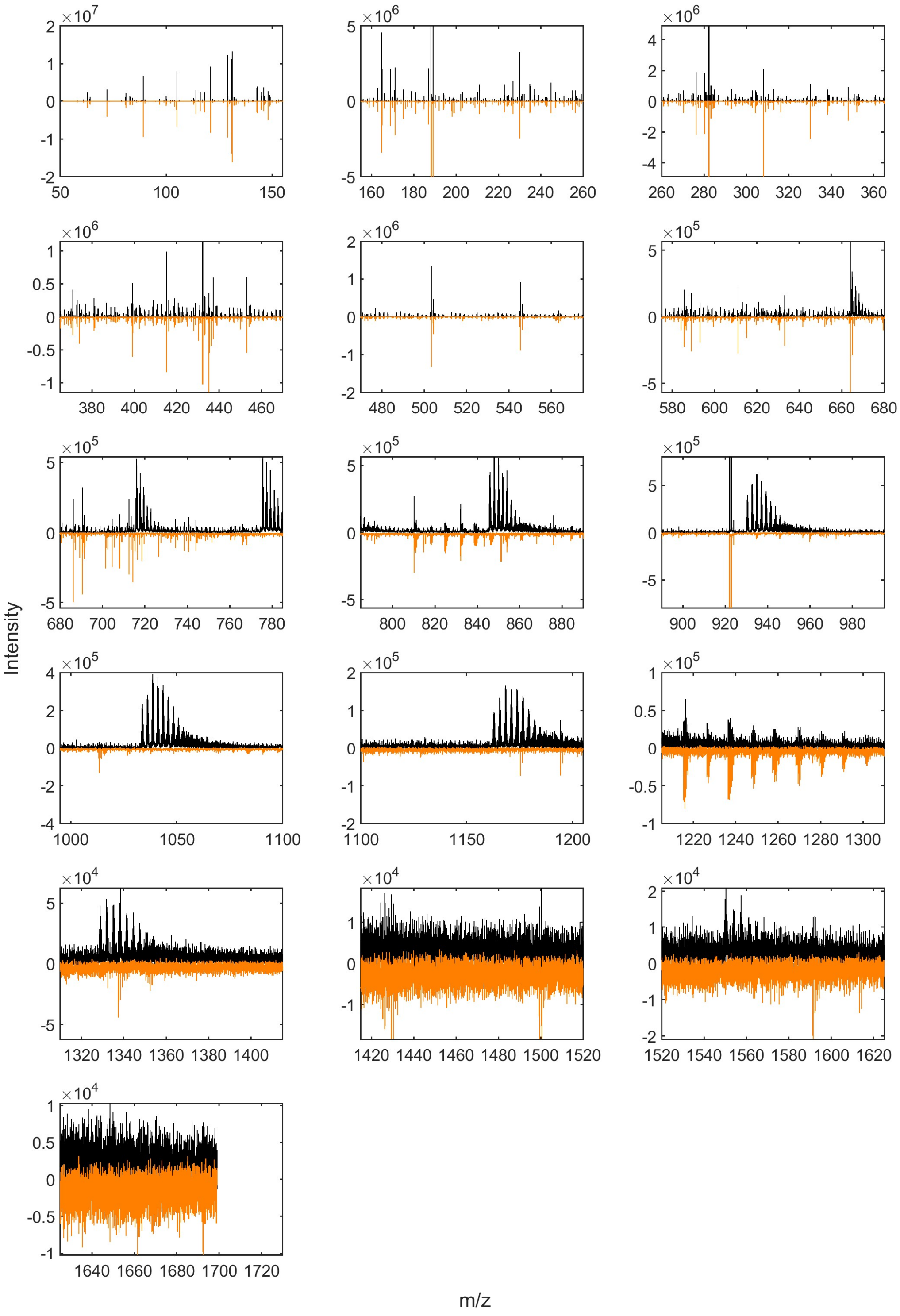
Intensity
m/z

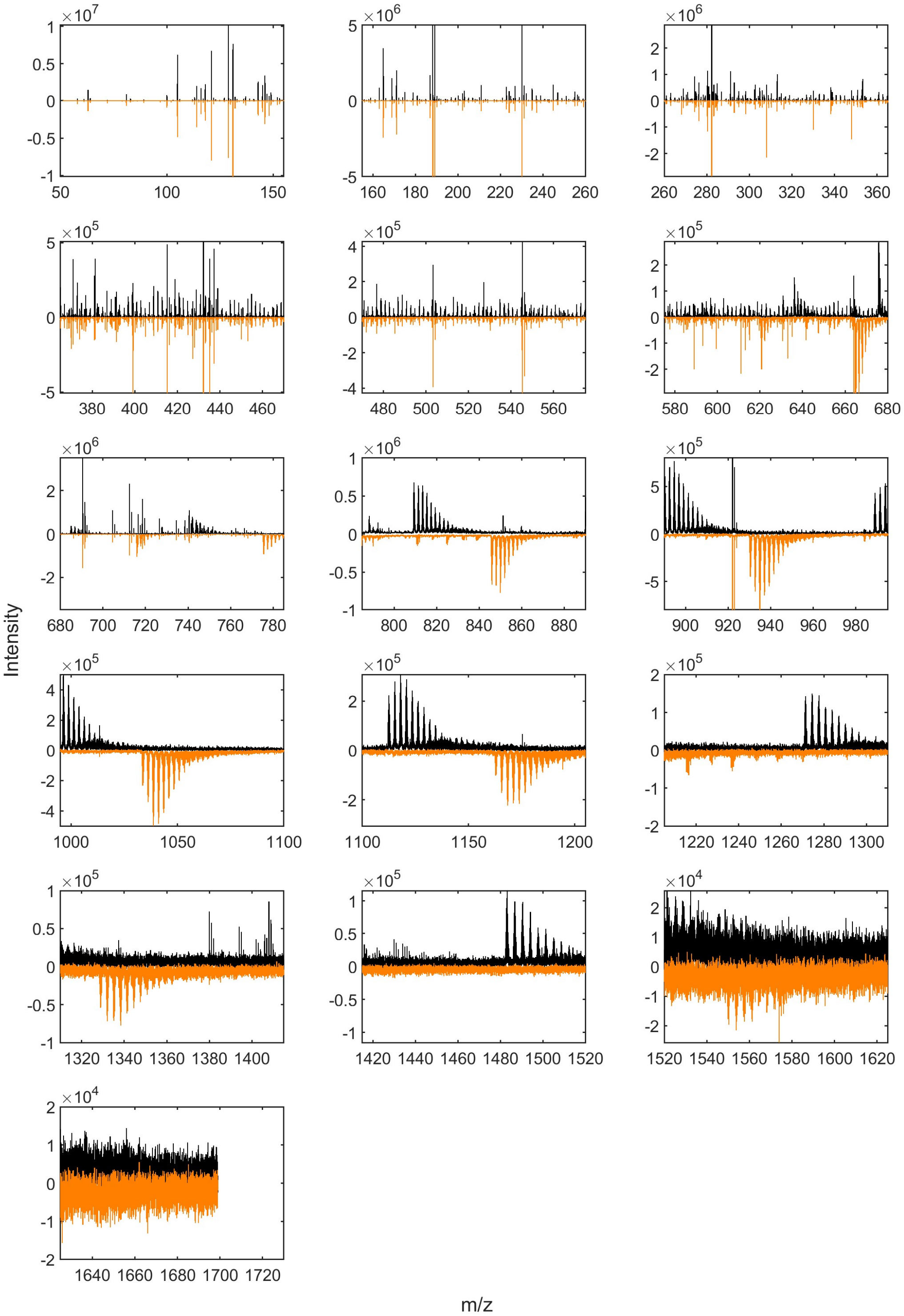
Intensity
m/z

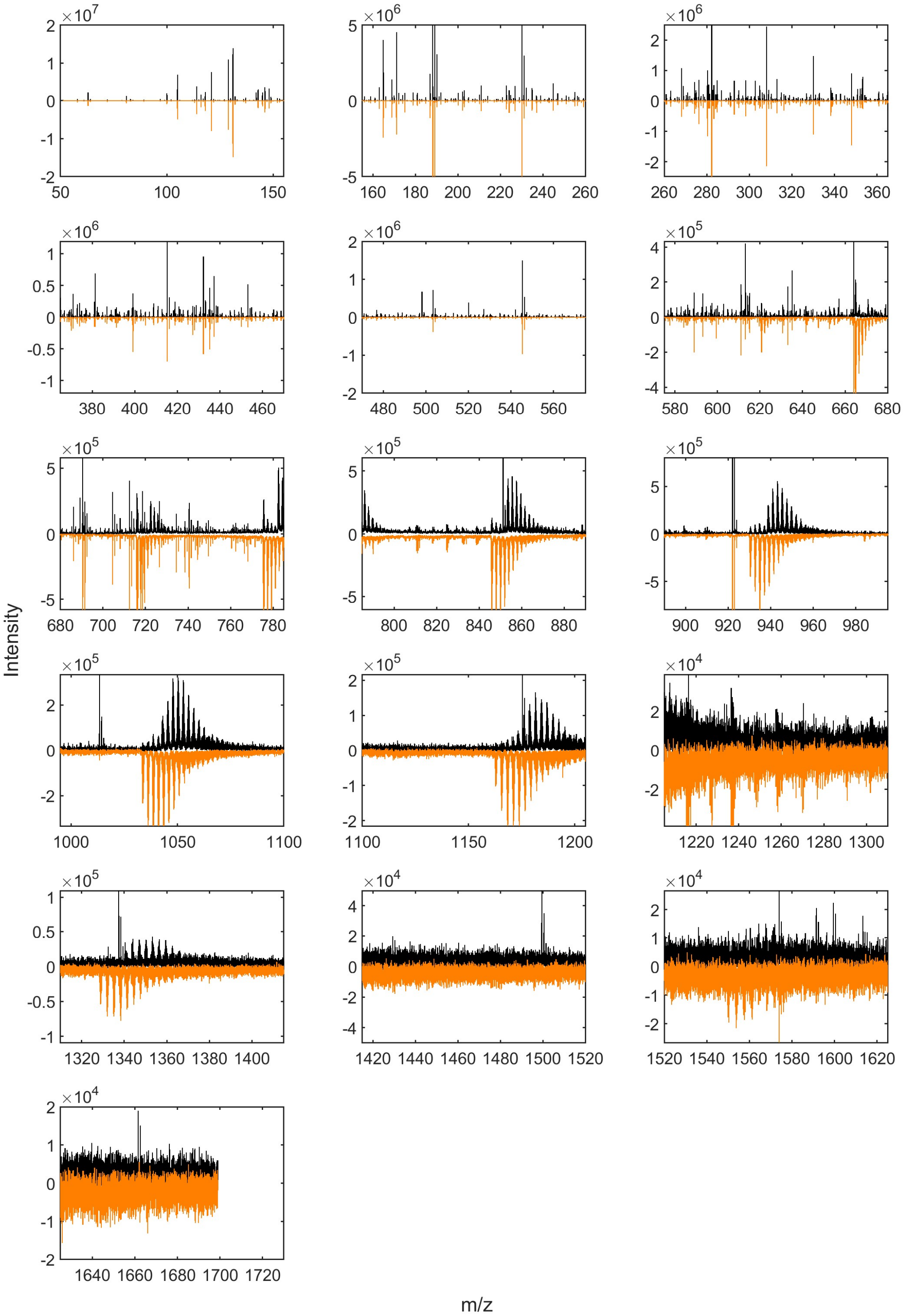

Intensity
m/z

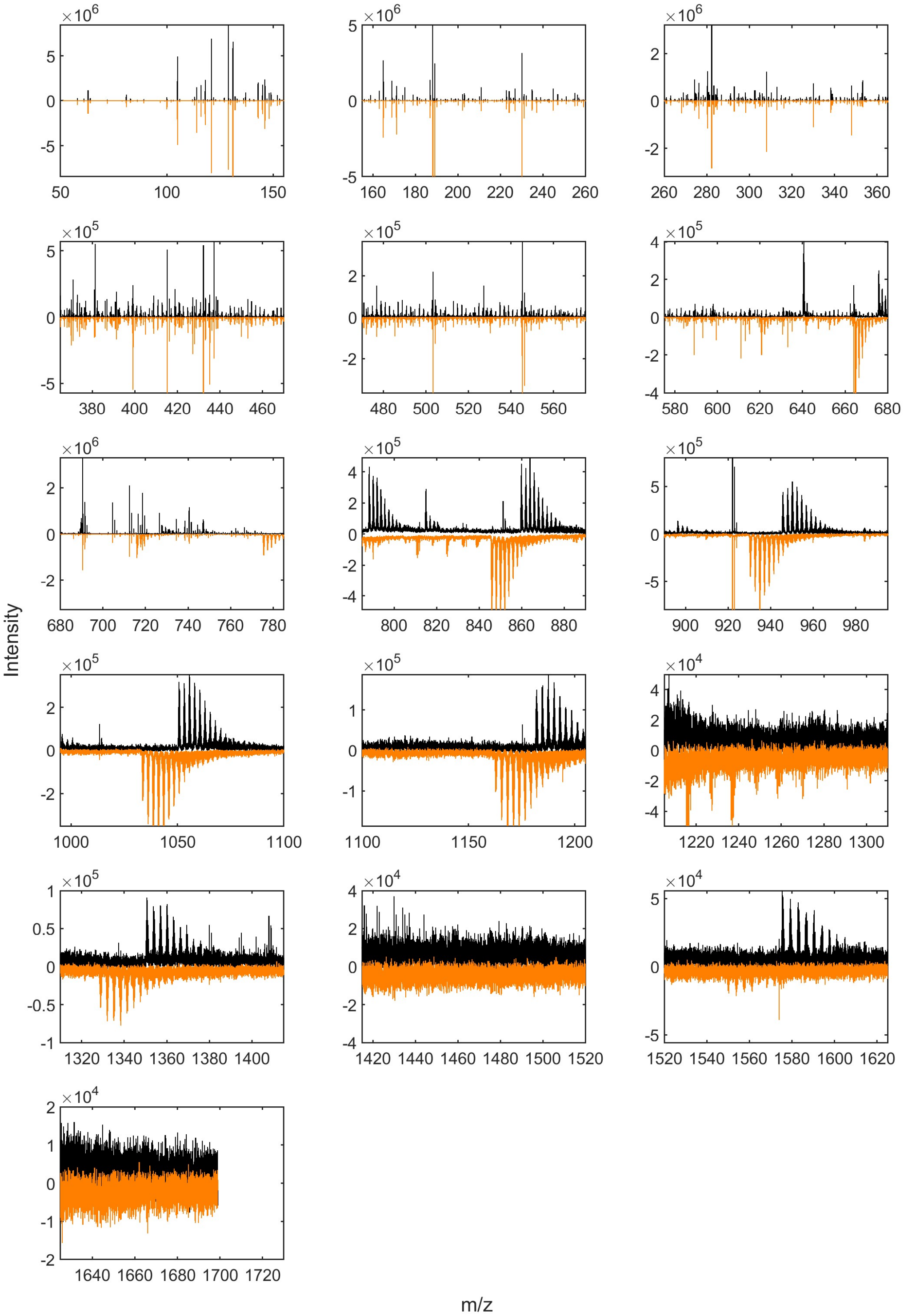
Intensity
m/z

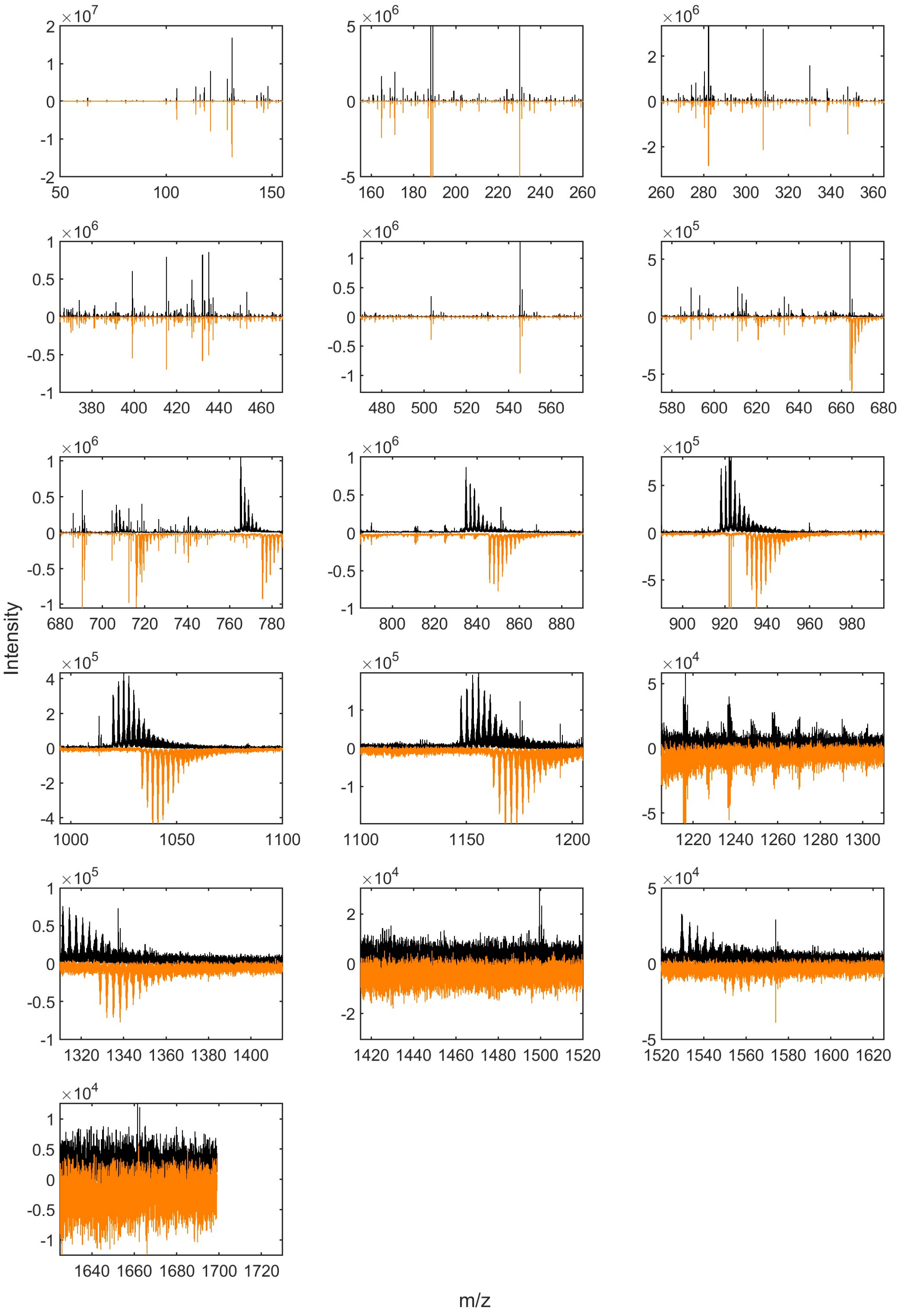
Intensity
m/z

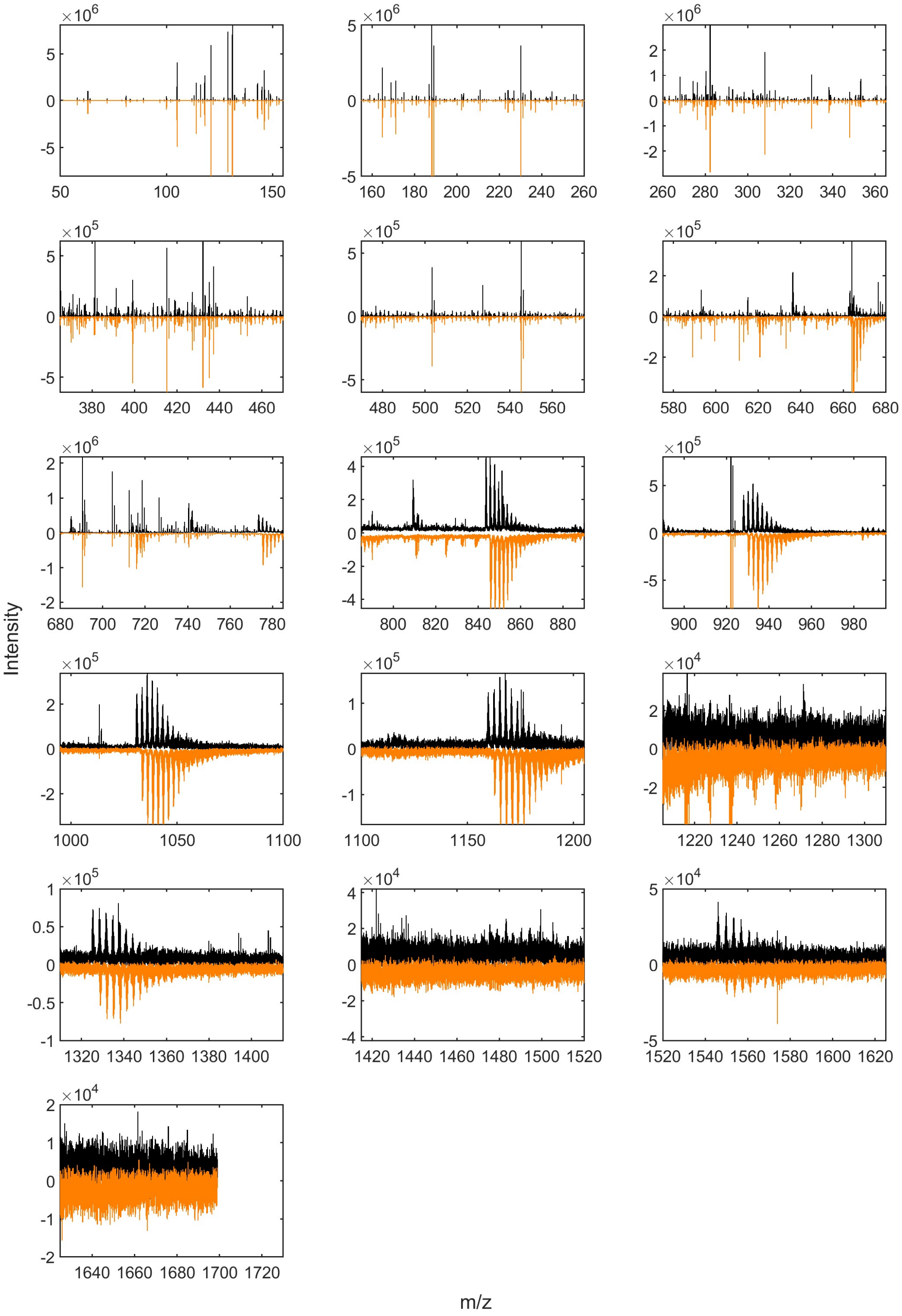
Intensity
m/z

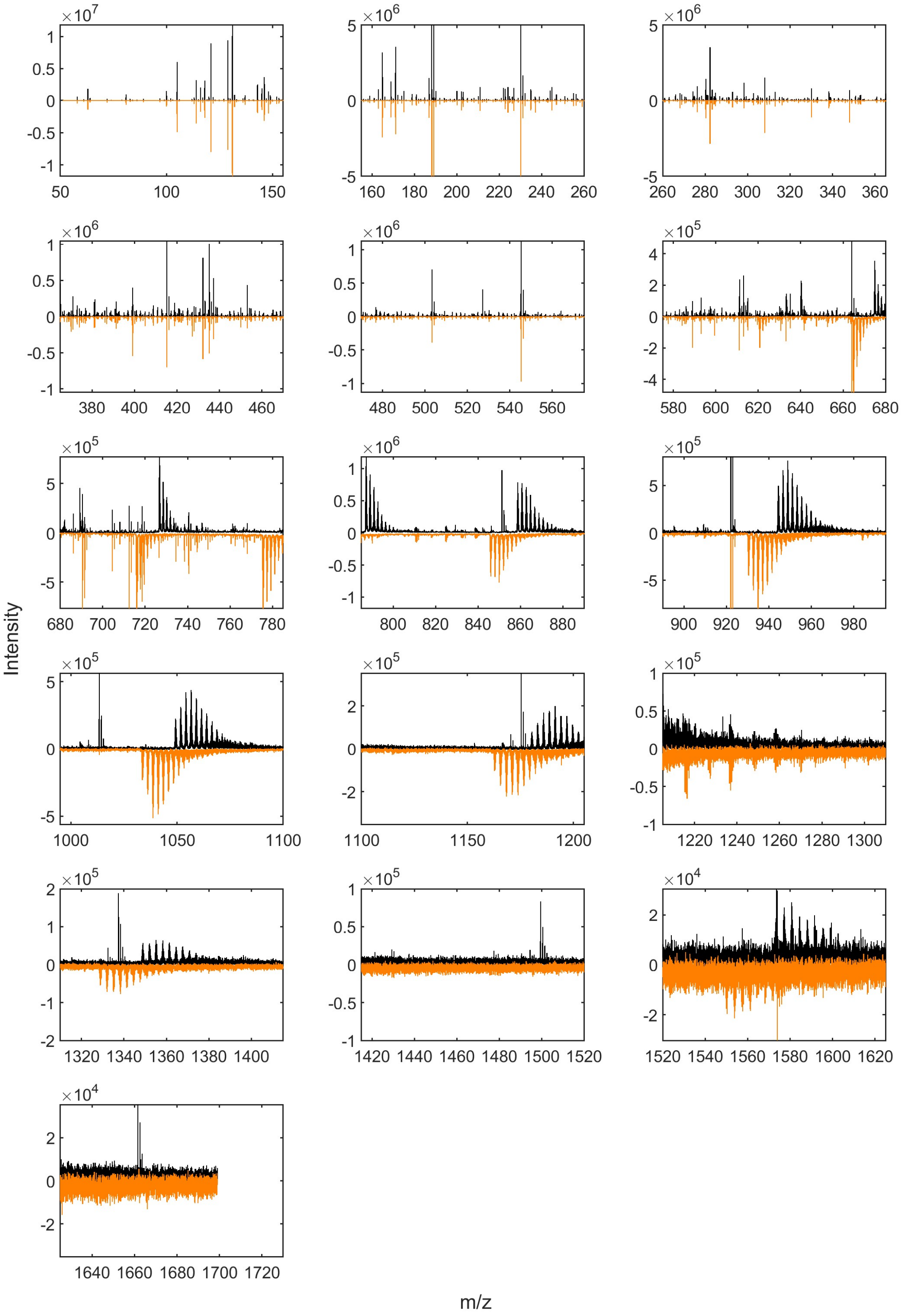
Intensity
m/z
