# Supplementary material for: Intact protein barcoding enables one-shot identification of CRISPRi strains and their metabolic state
Source: Cell Rep Methods. 2024 Nov 26;4(12):100908. doi: 10.1016/j.crmeth.2024.100908 (PMC11704613; doi:10.1016/j.crmeth.2024.100908)
Supplement: Document S2. Article plus supplemental information [file mmc8.pdf]

Cell Reports Methods

Report

Intact protein barcoding enables one-shot identification of CRISPRi strains and their metabolic state

Graphical abstract

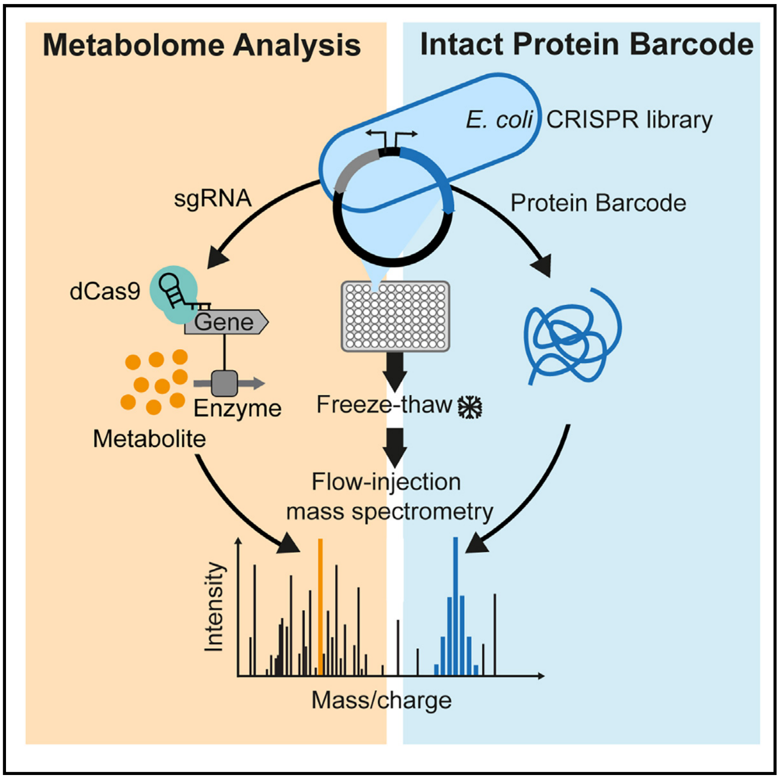

Authors

Vanessa Pahl, Paul Lubrano, Felicia Troßmann, Daniel Petras, Hannes Link

Correspondence

hannes.link@uni-tuebingen.de

In brief

Pahl et al. present a method called intact protein barcoding, which uses ubiquitin as a barcode to identify bacterial strains with mass spectrometry. The method can replace DNA sequencing of barcodes and enables strain identification and metabolome profiling in a single step.

Highlights

- Ubiquitin with N-terminal tags is a barcode readable by mass spectrometry
- Intact protein barcoding enables fast identification of CRISPRi strains by FI-MS
- We demonstrate simultaneous measurement of protein barcodes and metabolites
- The method is scalable to microtiter plates for high-throughput analysis

Pahl et al., 2024, Cell Reports Methods *4*, 100908
December 16, 2024 © 2024 The Author(s). Published by Elsevier Inc.
https://doi.org/10.1016/j.crmeth.2024.100908

CellPress

Cell Reports Methods

CellPress
OPEN ACCESS

Report

Intact protein barcoding enables one-shot identification of CRISPRi strains and their metabolic state

Vanessa Pahl,[1,2,5] Paul Lubrano,[1,2,3,5] Felicia Troßmann,[1,2,3] Daniel Petras,[2,4] and Hannes Link[1,2,3,6,*]
[1]Interfaculty Institute of Microbiology and Infection Medicine, University of Tübingen, Auf der Morgenstelle 24, 72076 Tübingen, Germany
[2]Cluster of Excellence "Controlling Microbes to Fight Infections", University of Tübingen, 72076 Tübingen, Germany
[3]M3 Research Center, University of Tübingen, 72076 Tübingen, Germany
[4]Department of Biochemistry, University of California, Riverside, 169 Aberdeen Dr., Riverside, CA 92507, USA
[5]These authors contributed equally
[6]Lead contact
*Correspondence: hannes.link@uni-tuebingen.de
https://doi.org/10.1016/j.crmeth.2024.100908

MOTIVATION Pooled cloning approaches with synthetic oligonucleotides can create large libraries of strain variants, but identifying individual strains requires DNA sequencing of strain-specific barcodes (e.g., guide RNAs in CRISPR libraries). The motivation behind this study was to develop a method that identifies protein-based barcodes with mass spectrometry. This method enables detection of barcodes and metabolites at the same time and thus improves the efficiency of metabolome screening in genetically engineered libraries.

SUMMARY

Detecting strain-specific barcodes with mass spectrometry can facilitate the screening of genetically engineered bacterial libraries. Here, we introduce intact protein barcoding, a method to measure protein-based library barcodes and metabolites using flow injection mass spectrometry (FI-MS). Protein barcodes are based on ubiquitin with N-terminal tags of six amino acids. We demonstrate that FI-MS detects intact ubiquitin proteins and identifies the mass of N-terminal barcodes. In the same analysis, we measured relative concentrations of primary metabolites. We constructed six ubiquitin-barcoded CRISPR interference (CRISPRi) strains targeting metabolic enzymes and analyzed their metabolic profiles and ubiquitin barcodes. FI-MS detected barcodes and distinct metabolome changes in CRISPRi-targeted pathways. We demonstrate the scalability of intact protein barcoding by measuring 132 ubiquitin barcodes in microtiter plates. These results show that intact protein barcoding enables fast and simultaneous detection of library barcodes and intracellular metabolites, opening up new possibilities for mass spectrometry-based barcoding.

INTRODUCTION

Rapid identification of bacterial strains within genetic libraries is important for synthetic biology applications, especially for screening of synthetic metabolic pathways and engineered enzymes. Traditional methods involve DNA barcoding, where unique DNA sequences are integrated into each bacterial strain. These sequences are then amplified and matched against a barcode database using high-throughput sequencing technologies.[1,2] DNA barcoding offers high specificity but lacks the capability to capture molecular phenotypes of engineered bacteria in the same measurement, because DNA barcoding is limited to inference of fitness phenotypes via barcode frequency. Alternatively, RNA barcodes can be visualized using fluorescence microscopy combined with fluorescence *in situ* hybridization to detect more complex phenotypes and for *in situ* genotyping of CRISPR libraries.[3] Recently, CRISPR screens have been combined with time-of-flight mass cytometry or immunohistochemistry for multiplexed cell barcoding at the protein level.[4,5] This method uses protein barcodes with triplet epitopes linked to specific guide RNAs for multidimensional protein profiling in single-cell pooled screens. Protein barcodes were also used for high-throughput screens of protein binding libraries using so-called flycodes and mass spectrometry.[6] However, there are currently no methods to simultaneously measure barcodes and metabolites. Such measurements could provide almost real-time strain identification and can significantly reduce the time and cost required for metabolome analyses of CRISPR libraries.

This is an open access article under the CC BY license (http://creativecommons.org/licenses/by/4.0/).

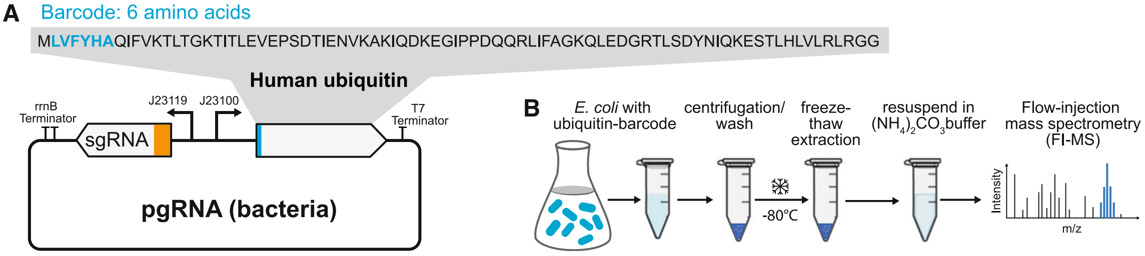

Figure 1. Protein barcoding with ubiquitin and detection by flow injection mass spectrometry
(A) The gene encoding human ubiquitin was integrated next to the sgRNA on the plasmid pgRNA-bacteria.[7] An N-terminal sequence of 6 amino acids serves as a barcode. Shown is the LVFYHA-ubiquitin barcode.
(B) Sampling protocol for simultaneous detection of metabolites and intact ubiquitin by flow injection mass spectrometry (FI-MS).

In this study, we address this limitation and introduce a method that uses high-throughput mass spectrometry to detect protein barcodes and metabolites in the same sample and in the same measurement.

RESULTS

As a case study for intact protein barcoding, we used CRISPR interference (CRISPRi) in *E. coli* metabolism. For CRISPRi, we used a catalytically dead Cas9 mutant to inhibit transcription of a target gene, which is defined by the base-pairing region of the single-guide RNA (sgRNA).[7] To label CRISPRi strains with a protein barcode, we co-expressed the sgRNA and a small protein from the same plasmid (Figure 1A). As the protein barcode, we selected human ubiquitin, a small protein consisting of 76 amino acids, which can be detected by intact protein analysis via top-down mass spectrometry.[8] To create unique barcodes, we engineered ubiquitin by adding a six-amino-acid tag to its N terminus (Figure 1A). Initially, we added a random sequence, LVFYHA, to ubiquitin, which resulted in the LVFYHA-ubiquitin barcode. This barcode was expressed under a constitutive promoter from the same plasmid as the sgRNA. Ubiquitin and sgRNA were oriented in opposite directions, with a 38 bp spacer that separates their respective promoter sequences. This design enables the exchange of sgRNA protospacer sequences and N-terminal tags using a single 241 bp DNA oligonucleotide (Figure 1A).

First, we confirmed expression of the LVFYHA-ubiquitin barcode in an *E. coli* CRISPRi strain, which carried a non-targeting sgRNA, and evaluated barcode detection by mass spectrometry. As a control strain, we used the same CRISPRi strain but without the ubiquitin barcode. Cell extracts were prepared from cultures in exponential growth phase using a single freeze-thaw cycle and resuspension in ammonium carbonate buffer to ensure intact protein recovery (Figure 1B). The cell extracts were then analyzed by flow injection mass spectrometry (FI-MS),[9,10] which has been shown to detect metabolite features and protein features in a single measurement.[11]

FI-MS detected 1,044 *m/z* features that matched theoretical masses of the LVFYHA-ubiquitin barcode, considering different charge states, sodium adducts, and 13-C isotopes (Table S1; Figure 2A). Importantly, 73% of these features were significantly more abundant in the strain that expressed LVFYHA-ubiquitin compared to the control strain ($p < 0.01$ and fold change > 4), thus demonstrating reproducible detection of the barcode across three replicates (Figure 2B). Most significant *m/z* features resulted from the LVFYHA-ubiquitin species that had 9 and 10 positive charges, which are within an *m/z* range of 930–1,050 (Figure 2B). No significant features were detected in the lower mass range below 600, which is the mass range of most primary metabolites, and therefore we assumed that ubiquitin does not interfere with metabolite detection (Figure 2A; Data S1). Finally, we used the FLASHdeconv tool[12] for spectral deconvolution of the FI-MS data. This analysis accurately identified the monoisotopic mass of LVFYHA-ubiquitin at 9,290.00 Da (Figure 2C). In summary, these results demonstrate that ubiquitin with an N-terminal tag is expressed in *E. coli* and that this barcode can be effectively identified with FI-MS and a fast extraction protocol that does not require protein digestion into peptides.

Next, we created six additional ubiquitin barcodes, each with a distinct mass, and tested whether we can distinguish these barcodes based on their mass alone (Figure 3A). The six barcodes were combined with sgRNAs targeting genes that encode enzymes of different metabolic pathways (Figure 3B). Four of the target genes encoded enzymes in biosynthesis pathways of the amino acids histidine (*hisD*), lysine (*dapE*), threonine (*thrC*), and leucine (*leuB*). Two other target-genes encoded isocitrate dehydrogenase (*icd*) in the tricarboxylic acid (TCA) cycle and 1-deoxy-D-xylulose 5-phosphate reductoisomerase (*dxr*) in the methylerythritol phosphate (MEP) pathway. The control strain was again the LVFYHA-ubiquitin barcode in combination with a non-targeting sgRNA.

Metabolites and proteins of these strains were sampled after 6.5 h of growth in minimal glucose medium and anydrotetracycline to induce the CRISPRi system. FI-MS analysis of the extracts showed distinct MS1 spectra with the characteristic clusters of *m/z* features, originating from 13-C isotopes and sodium adducts (Data S1). In four strains (*dxr*, *icd*, *hisD*, and *thrC*), the 5 most abundant monoisotopic masses matched the theoretical monoisotopic mass of the respective barcode and up to four sodium adducts (Figure S1; Table S2). Few signals determined by spectral deconvolution differed by 1 Da, presumably due to errors in assignment of either charge states or isotope distribution during deconvolution. In two strains (*dapE* and *leuB*), spectral deconvolution did not result in the correct monoisotopic mass of the respective barcodes (SIYFLD and SGGSGN). In both

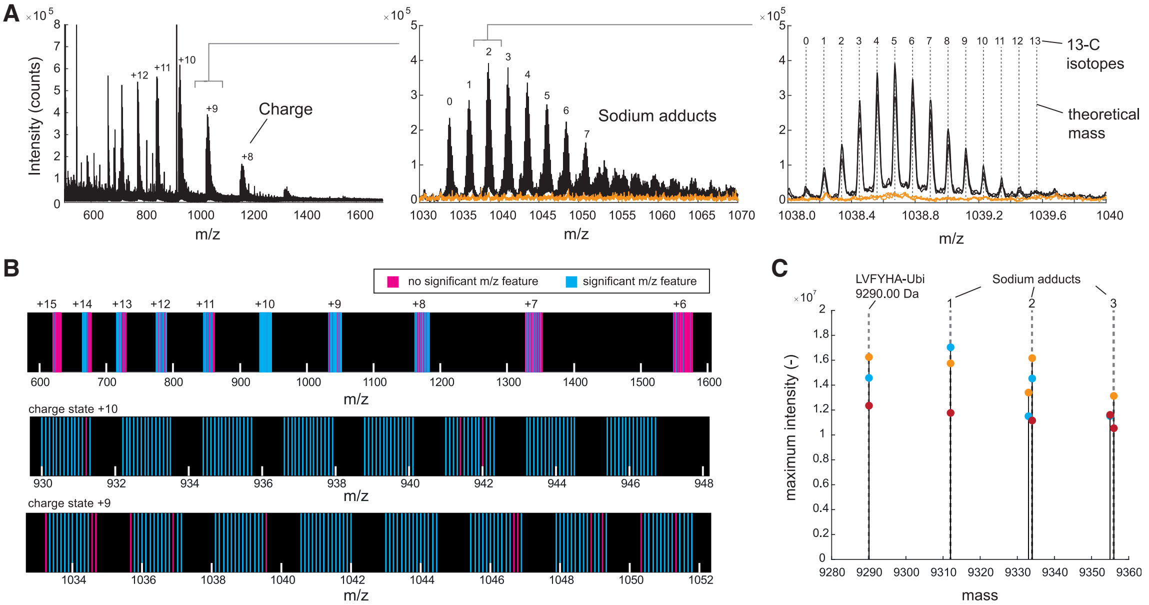

Figure 2. FI-MS detects the LVFYHA-ubiquitin barcode

(A) Mass spectra of cell extracts from *E. coli* expressing LVFYHA-ubiquitin (black). Extracts from a control strain without ubiquitin are shown in orange. Shown is the MS1 spectrum in positive mode from 500 to 1,700 *m/z* measured with FI-MS. Different charge states are due to varying degrees of protonation; z = 8–12 are indicated (left). Sodium adducts of the +9 charge state; 0–7 sodium adducts are indicated (center). 13-C isotope distribution of z = +9 charge state and 2 sodium adducts; exact monoisotopic masses are shown as dashed lines (right).

(B) *m/z* features were annotated to exact monoisotopic masses of LVFYHA-ubiquitin with different charges, sodium adducts, and 13-C isotopes. Annotated *m/z* features that were significantly different in LVFYHA-ubiquitin samples relative to the control samples are shown in blue ($n = 3$ samples, $p < 0.01$, fold change > 4).

(C) Spectral deconvolution of MS1 spectra of LVFYHA-ubiquitin identified the exact monoisotopic mass of LVFYHA-ubiquitin (9,290.00) and sodium adducts. Shown are 5 monoisotopic masses with a highest summed intensity of 3 replicates. Dashed lines are exact masses, and dots with different colors are deconvoluted masses of 3 replicates.

cases, the mass difference between measured and theoretical mass was −131 Da, which corresponds to the mass of a peptide-bound methionine. This suggested hydrolytic cleavage of the N-terminal methionine from the SIYFLD-ubiquitin and SGGSGN-ubiquitin barcodes. In *E. coli*, the enzyme methionine aminopeptidase catalyzes the removal of the first methionine residue in proteins, and its activity is higher when the first amino acid has small side chains,[13] such as the serine of the SIYFLD-ubiquitin and SGGSGN-ubiquitin barcodes. Consequently, the design of the ubiquitin barcodes could be optimized by selecting amino acids with larger side chains at the beginning of the barcode to avoid methionine cleavage.

Having established that FI-MS identifies ubiquitin barcodes, we investigated whether we could detect metabolites in the same FI-MS data. For FI-MS analysis, each sample is injected twice consecutively under constant settings: once in positive ionization mode and once in negative ionization mode. MS1 spectra are recorded for 30 s following each injection. Protein barcodes are detected in positive ionization mode, whereas metabolites are detected in both ionization modes. FI-MS detected 523 *m/z* features that we could annotate to 698 metabolites in the genome-scale metabolic model of *E. coli*, *i*ML1515[14] (Table S3). Principal-component analysis separated the different CRISPRi strains (Figure 3C), thus indicating that our method is robust and captures distinct metabolic states induced by the CRISPRi perturbations. Next, we inspected metabolites in the metabolic pathways that are associated with the CRISPRi perturbation, which are biosynthesis pathways of histidine, leucine, threonine, and lysine; the MEP pathway and the TCA cycle (Figure 3D). The *hisD* strain showed strong increases (fold change > 8) of 5 intermediates in the histidine pathways, including the *hisD* substrate metabolite histidinol (histd). This increase of histidine intermediates is likely caused by a bottleneck at the end of the pathway through knockdown of *hisD*. Similarly, FI-MS detected an accumulation of substrate metabolites of the other three CRISPRi strains that target amino acids pathways: N-succinyl-L-2,6-diaminoheptanedioate in the *dapE* strain, O-phospho-L-homoserine in the *thrC* strain, and (2R,3S)-3-isopropylmalate in the *leuB* strain. Furthermore, the substrate metabolites of the *icd* and dxr strain, isocitrate and 1-deoxy-D-xylulose 5-phosphate, increased. These results show that our approach detects distinct metabolome changes in the CRISPRi strains, including stronger metabolome changes in the pathways targeted by CRISPRi (e.g., increase of substrate metabolites in all 6 CRISPRi strains).

To further assess the scalability of our intact protein barcoding approach, we created a larger library arrayed in 96-well plates. We designed 500 unique DNA oligonucleotides, each encoding a distinct 6-amino-acid tag on ubiquitin and a sgRNA, resulting in 500 unique protein barcodes. The amino acid sequences of these barcodes were designed so that each barcode had a different mass that differed by 1 Da from its nearest

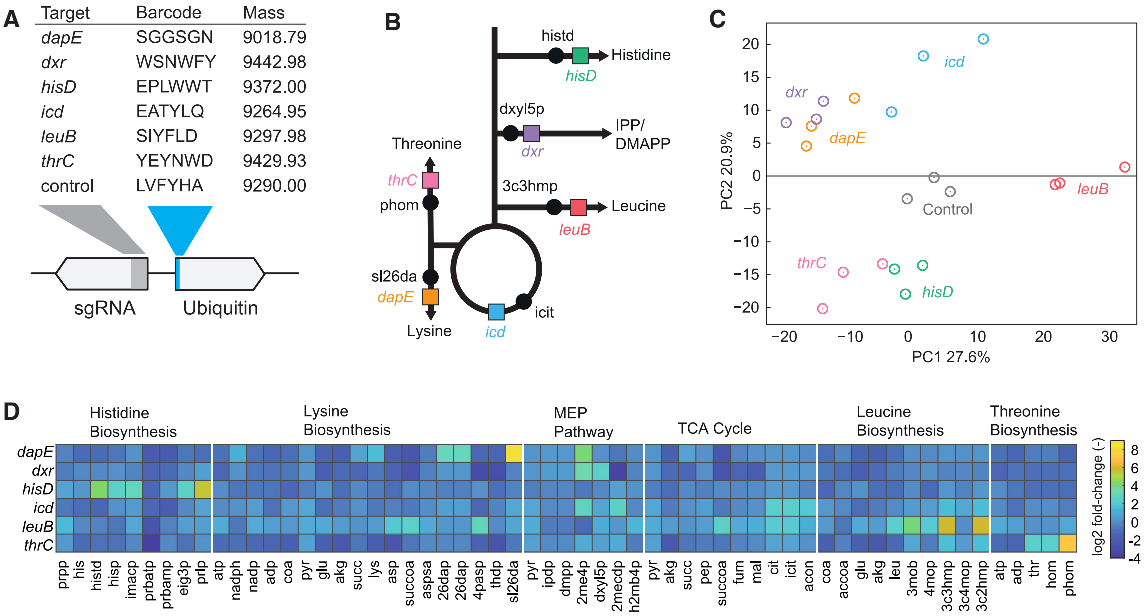

Figure 3. Ubiquitin barcodes of 6 CRISPRi strains allow simultaneous analysis of the metabolic signature of CRISPRi strains

(A) CRISPRi strains. Shown is the gene targeted by the sgRNA and the 6 amino acids of the ubiquitin barcode. The monoisotopic mass of the ubiquitin barcode is given in Dalton.

(B) CRISPRi target pathways. Shown are the target enzymes and their substrate metabolites.

(C) Principal-component analysis of *m/z* features that were annotated to metabolites. Shown are PC1 and PC2 of 698 metabolites measured in 3 samples of 7 CRISPRi strains.

(D) Levels of metabolites in metabolic pathways that are targeted by the six CRISPRi strains. Metabolite levels are shown as log2 fold change relative to the control strain with a non-targeting sgRNA. The heatmap shows the mean from 3 cultures. Metabolites are grouped by pathways.

prpp, 5-phospho-alpha-D-ribose 1-diphosphate; his, L-histidine; histd, histidinol; hisp, L-histidinol phosphate; imacp, 3-(imidazol-4-yl)-2-oxopropyl phosphate; prbatp, 1-(5-phosphoribosyl)-ATP; prbamp, 1-(5-phosphoribosyl)-AMP; eig3p, D-erythro-1-(imidazol-4-yl)glycerol 3-phosphate; prlp, 5-[(5-phospho-1-deoxyribulos-1-ylamino)methylideneamino]-1-(5-phosphoribosyl)imidazole-4-carboxamide; nadph, nicotinamide adenine dinucleotide phosphate (reduced); nadp, nicotinamide adenine dinucleotide phosphate; coa, coenzyme A; pyr, pyruvate; glu, L-glutamate; akg, 2-oxoglutarate; succ, succinate; lys, L-lysine; asp, L-aspartate.

neighbor. The 1-Da mass difference was used to ensure sufficient differentiation of barcodes in the MS1 spectra. The library was constructed using a pooled cloning approach with synthetic DNA oligonucleotides that contained the sgRNA and the N-terminal tag of ubiquitin. From the pooled library, 190 random isolates where sorted into two 96-well plates. Sequencing revealed that 132 of 190 strains (69%) had a correct barcode (Figure 4A; Table S3). However, only 67 strains had the correct combination of barcode and sgRNA, as in the originally designed library. This discrepancy is likely due to a known shuffling effect that can occur during the construction of pooled libraries.[15,16]

To test whether we can detect barcodes with higher throughput, we used deep-well plates to cultivate the strains and extracted protein barcodes by centrifugation and a freeze-thaw cycle (Figure 4A). The barcodes were analyzed using FI-MS, followed by spectral deconvolution with FLASHDeconv. We compared the five monoisotopic masses with the highest intensities against the theoretical barcode masses. In 97 of 132 strains with a correct barcode (based on sequencing), we detected the barcode with FI-MS (Figure 4B; Table S4). After deconvolution, the correct monoisotopic mass was the highest-intensity peak in 48 of 132 strains (36%). In 7 strains, the correct barcode was detected among the top five most intense masses, although not as the highest, due to the presence of sodium adducts. Additionally, in 38 strains (29%), the highest intensity peak corresponded to a mass −131 Da from the theoretical mass, indicating cleavage of the N-terminal methionine. For 4 of these cases (3%), the correct barcode with −131 Da was among the top five masses but not the highest, which was again due to sodium adducts. Thus, in total, we successfully detected the correct barcode signal in 97 of 132 strains (73%), demonstrating that intact protein barcoding is feasible even with a larger and more complex library. We further inspected the mass differences between the measured and theoretical barcode masses, showing that the average error was only 0.03 Da, and no barcode had errors >0.05 Da (Figure S2A). This low error suggests that the spacing between barcodes could be reduced by at least 10-fold compared to the current library to increase the number of barcode libraries in future applications. Of the 132 barcodes analyzed, 35 were not identified using FI-MS, and 32 of them showed low intensities in the MS1 data (Figure 4C). Matched barcodes, in contrast, had, on average, high intensities. This suggests that the failure of barcode detection could be caused by poor protein expression or inefficient ionization during top-down mass spectrometry.

CellPress
OPEN ACCESS

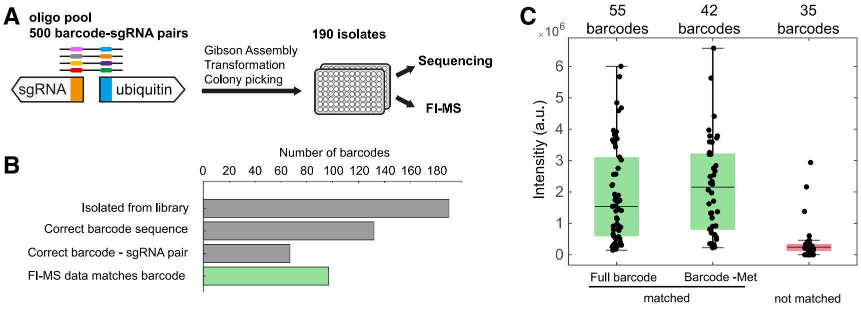

Figure 4. High-throughput analysis of ubiquitin barcodes in microtiter plates

(A) A pooled CRISPRi library with 500 ubiquitin barcodes was constructed with synthetic DNA oligos (Table S5). 190 of the strains were randomly picked, transferred to 96-well plates, and analyzed by sequencing and FI-MS.

(B) Sequencing showed that 132 strains had correct barcodes. FI-MS detected 97 of the barcodes.

(C) Summed intensity after deconvolution of 132 barcodes that had a correct barcode sequence. Barcodes are grouped into those identified by FI-MS (full barcode matched), barcodes with methionine (Met) cleavage that matched in the FI-MS data (barcode-Met matched), and barcodes that did not match the FI-MS data (not matched).

DISCUSSION

In this study, we introduced intact protein barcoding as an approach for barcoding CRISPRi strains using ubiquitin, which is integrated with the sgRNA in a dual expression system. Our method combines aqueous extraction and MS1 spectral analysis, which efficiently captures both protein barcodes and primary metabolites. Apart from CRISPRi strains, the approach should enable rapid identification of other libraries of engineered bacteria, and it has advantages over traditional methods that use plasmid extraction and sequencing. The method could identify unique barcode signatures and concentration changes across hundreds of metabolites within 1-min analysis time per sample. Apart from CRISPRi libraries, future applications could create enzyme variant libraries, where intact protein barcoding could be used to rapidly screen for specific metabolites produced by different enzyme variants or to assess activity changes.

Automated colony picking and high-throughput sampling in microtiter plates is probably the most effective method to increase the throughput of our technology. However, it is also possible to skip the extraction step, as we identified the LVFYHA-ubiquitin barcode by injecting whole living cells directly into the mass spectrometer (Figure S2B). Thus, future studies could measure intact protein barcodes with real-time metabolomics[15] or imaging mass spectrometry with MALDI time-of-flight mass spectrometry applied directly to microbes on agar-based medium[16] to screen metabolic states of CRISPR libraries with ultra-high throughput. Although MALDI time-of-flight mass spectrometry could enhance the sensitivity of barcode detection, it would likely reduce metabolite detection compared to electrospray ionization time-of-flight mass spectrometry used in this study.

We used a relatively small set of 500 protein tags for this feasibility study, but the potential of this method may extend far beyond this initial scale. With the 0.03 Da accuracy that we reached in our setup, it should be possible to differentiate more than 500 barcodes at the MS1 level. However, at the MS/MS level, even isobaric tags (barcodes with identical masses but different sequences) can be differentiated based on their fragmentation patterns. This would open up the possibility of creating and distinguishing thousands of unique protein barcodes. As a result, we anticipate that our method could serve as a starting point for the further development of MS/MS-based differentiation of sequence tags, leading to ultra-high-throughput metabolome screening in large libraries of engineered bacteria. This advancement could have broad and significant applications in synthetic biology, enabling scalable analyses of genetic libraries only with mass spectrometry.

Limitations of the study

While our results demonstrate the potential of intact protein barcoding for high-throughput identification and metabolic profiling of bacterial strains, the full potential of the method is limited by achieving correct sgRNA-barcode combinations with pooled cloning approaches. Currently, the primary challenge is the shuffling effect that we observed during library construction and errors introduced during cloning, which is a known problem[17,18] and resulted in strains containing incorrect sgRNA-barcode pairs. In this study, we validated our method by sequencing the library to confirm the sgRNA-barcode combinations. However, in future applications of this method, sequencing will not be required once the pooled library construction process is optimized. Additionally, our data show that 26% of barcodes do not produce a detectable signal, which limits the overall effectiveness of the method. This problem could be mitigated by optimizing the design principles of the barcodes through large-scale measurements of diverse barcode sequences to better understand the factors influencing signal strength.

RESOURCE AVAILABILITY

Lead contact

Requests for further information, resources, and reagents should be directed to and will be fulfilled by the lead contact, Hannes Link (hannes.link@uni-tuebingen.de).

Materials availability

Plasmids and strains generated in this study are available upon request from the lead contact, Hannes Link (hannes.link@uni-tuebingen.de).

Data and code availability

- FI-MS source data have been deposited at MassIVE: MSV000096254 (https://massive.ucsd.edu/ProteoSAFe/dataset.jsp?accession=MSV000096254). Scripts used to generate the figures presented in this paper are available from the lead contact upon request.
- This study does not report original code.
- Any additional information required to reproduce this work is available from the lead contact.

ACKNOWLEDGMENTS

This work was funded by the DFG Cluster of Excellence EXC2124 “Controlling Microbes to Fight Infection” (CMFI).

AUTHOR CONTRIBUTIONS

Conceptualization, all authors; methodology, all authors; investigation, all authors; visualization, V.P. and H.L.; funding acquisition, D.P. and H.L.; project administration, H.L.; supervision, H.L.; writing – original draft, V.P. and H.L.

DECLARATION OF INTERESTS

The authors declare no competing interests.

STAR★METHODS

Detailed methods are provided in the online version of this paper and include the following:

- KEY RESOURCES TABLE
- EXPERIMENTAL MODEL AND STUDY PARTICIPANT DETAILS
  - Strains and culture
  - Media
- METHOD DETAILS
  - Cloning of pgRNA-ubiquitin
  - Cloning of CRISPRi strains
  - Cultivation and sampling of ubiquitin-barcodes and metabolites in shake flasks
  - Cultivation and sampling of ubiquitin-barcodes in 96-well plates
  - Flow-injection mass spectrometry (FI-MS)
- QUANTIFICATION AND STATISTICAL ANALYSIS

SUPPLEMENTAL INFORMATION

Supplemental information can be found online at https://doi.org/10.1016/j.crmeth.2024.100908.

Received: June 11, 2024
Revised: October 9, 2024
Accepted: November 4, 2024
Published: November 26, 2024

REFERENCES

1. Shalem, O., Sanjana, N.E., and Zhang, F. (2015). High-throughput functional genomics using CRISPR–Cas9. Nat. Rev. Genet. *16*, 299–311. https://doi.org/10.1038/nrg3899.
2. Shiver, A.L., Culver, R., Deutschbauer, A.M., and Huang, K.C. (2021). Rapid ordering of barcoded transposon insertion libraries of anaerobic bacteria. Nat. Protoc. *16*, 3049–3071. https://doi.org/10.1038/s41596-021-00531-3.
3. Lawson, M.J., Camsund, D., Larsson, J., Baltekin, Ö., Fange, D., and Elf, J. (2017). In situ genotyping of a pooled strain library after characterizing complex phenotypes. Mol. Syst. Biol. *13*, 947. https://doi.org/10.15252/msb.20177951.
4. Wroblewska, A., Dhainaut, M., Ben-Zvi, B., Rose, S.A., Park, E.S., Amir, E.-A.D., Bektesevic, A., Baccarini, A., Merad, M., Rahman, A.H., and Brown, B.D. (2018). Protein Barcodes Enable High-Dimensional Single-Cell CRISPR Screens. Cell *175*, 1141–1155.e16. https://doi.org/10.1016/j.cell.2018.09.022.
5. Dhainaut, M., Rose, S.A., Akturk, G., Wroblewska, A., Nielsen, S.R., Park, E.S., Buckup, M., Roudko, V., Pia, L., Sweeney, R., et al. (2022). Spatial CRISPR genomics identifies regulators of the tumor microenvironment. Cell *185*, 1223–1239.e20. https://doi.org/10.1016/j.cell.2022.02.015.
6. Egloff, P., Zimmermann, I., Arnold, F.M., Hutter, C.A.J., Morger, D., Opitz, L., Poveda, L., Keserue, H.-A., Panse, C., Roschitzki, B., and Seeger, M.A. (2019). Engineered Peptide Barcodes for In-Depth Analyses of Binding Protein Ensembles. Nat. Methods *16*, 421–428. https://doi.org/10.1038/s41592-019-0389-8.
7. Qi, L.S., Larson, M.H., Gilbert, L.A., Doudna, J.A., Weissman, J.S., Arkin, A.P., and Lim, W.A. (2013). Repurposing CRISPR as an RNA-guided platform for sequence-specific control of gene expression. Cell *152*, 1173–1183. https://doi.org/10.1016/j.cell.2013.02.022.
8. Donnelly, D.P., Rawlins, C.M., DeHart, C.J., Fornelli, L., Schachner, L.F., Lin, Z., Lippens, J.L., Aluri, K.C., Sarin, R., Chen, B., et al. (2019). Best practices and benchmarks for intact protein analysis for top-down mass spectrometry. Nat. Methods *16*, 587–594. https://doi.org/10.1038/s41592-019-0457-0.
9. Fuhrer, T., Heer, D., Begemann, B., and Zamboni, N. (2011). High-throughput, accurate mass metabolome profiling of cellular extracts by flow injection-time-of-flight mass spectrometry. Anal. Chem. *83*, 7074–7080. https://doi.org/10.1021/ac201267k.
10. Farke, N., Schramm, T., Verhülsdonk, A., Rapp, J., and Link, H. (2023). Systematic analysis of in-source modifications of primary metabolites during flow-injection time-of-flight mass spectrometry. Anal. Biochem. *664*, 115036. https://doi.org/10.1016/j.ab.2023.115036.
11. Jiang, Y., Salladay-Perez, I., Momenzadeh, A., Covarrubias, A.J., and Meyer, J.G. (2023). Simultaneous Multi-Omics Analysis by Direct Infusion Mass Spectrometry (SMAD-MS). Preprint at bioRxiv. https://doi.org/10.1101/2023.06.26.546628.
12. Jeong, K., Kim, J., Gaikwad, M., Hidayah, S.N., Heikaus, L., Schlüter, H., and Kohlbacher, O. (2020). FLASHDeconv: Ultrafast, High-Quality Feature Deconvolution for Top-Down Proteomics. Cell Syst. *10*, 213–218.e6. https://doi.org/10.1016/j.cels.2020.01.003.
13. Xiao, Q., Zhang, F., Nacev, B.A., Liu, J.O., and Pei, D. (2010). Protein N-Terminal Processing: Substrate Specificity of Escherichia coli and Human Methionine Aminopeptidases. Biochemistry *49*, 5588–5599. https://doi.org/10.1021/bi1005464.
14. Monk, J.M., Lloyd, C.J., Brunk, E., Mih, N., Sastry, A., King, Z., Takeuchi, R., Nomura, W., Zhang, Z., Mori, H., et al. (2017). iML1515, a knowledgebase that computes Escherichia coli traits. Nat. Biotechnol. *35*, 904–908. https://doi.org/10.1038/nbt.3956.
15. Link, H., Fuhrer, T., Gerosa, L., Zamboni, N., and Sauer, U. (2015). Real-time metabolome profiling of the metabolic switch between starvation and growth. Nat. Methods *12*, 1091–1097. https://doi.org/10.1038/nmeth.3584.
16. Yang, J.Y., Phelan, V.V., Simkovsky, R., Watrous, J.D., Trial, R.M., Fleming, T.C., Wenter, R., Moore, B.S., Golden, S.S., Pogliano, K., and Dorrestein, P.C. (2012). Primer on Agar-Based Microbial Imaging Mass Spectrometry. J. Bacteriol. *194*, 6023–6028. https://doi.org/10.1128/jb.00823-12.
17. Xie, S., Cooley, A., Armendariz, D., Zhou, P., and Hon, G.C. (2018). Frequent sgRNA-barcode recombination in single-cell perturbation assays. PLoS One *13*, e0198635. https://doi.org/10.1371/journal.pone.0198635.
18. Hegde, M., Strand, C., Hanna, R.E., and Doench, J.G. (2018). Uncoupling of sgRNAs from their associated barcodes during PCR amplification of combinatorial CRISPR screens. PLoS One *13*, e0197547. https://doi.org/10.1371/journal.pone.0197547.
19. Brzovic, P.S., Lissounov, A., Christensen, D.E., Hoyt, D.W., and Klevit, R.E. (2006). A UbcH5/ubiquitin noncovalent complex is required for processive BRCA1-directed ubiquitination. Mol. Cell *21*, 873–880. https://doi.org/10.1016/j.molcel.2006.02.008.
20. Engler, C., Kandzia, R., and Marillonnet, S. (2008). A one pot, one step, precision cloning method with high throughput capability. PLoS One *3*, e3647. https://doi.org/10.1371/journal.pone.0003647.
21. García-Nafría, J., Watson, J.F., and Greger, I.H. (2016). IVA cloning: A single-tube universal cloning system exploiting bacterial In Vivo Assembly. Sci. Rep. *6*, 27459. https://doi.org/10.1038/srep27459.
22. Chung, C.T., Niemela, S.L., and Miller, R.H. (1989). One-step preparation of competent Escherichia coli: transformation and storage of bacterial

CellPress
OPEN ACCESS

cells in the same solution. Proc. Natl. Acad. Sci. USA *86*, 2172–2175. https://doi.org/10.1073/pnas.86.7.2172.

23. Donati, S., Kuntz, M., Pahl, V., Farke, N., Beuter, D., Glatter, T., Gomes-Filho, J.V., Randau, L., Wang, C.-Y., and Link, H. (2021). Multi-omics Analysis of CRISPRi-Knockdowns Identifies Mechanisms that Buffer Decreases of Enzymes in E. coli Metabolism. Cell Syst. *12*, 56–67.e6. https://doi.org/10.1016/j.cels.2020.10.011.

24. Gibson, D.G., Young, L., Chuang, R.-Y., Venter, J.C., Hutchison, C.A., and Smith, H.O. (2009). Enzymatic assembly of DNA molecules up to several hundred kilobases. Nat. Methods *6*, 343–345. https://doi.org/10.1038/nmeth.1318.

STAR★METHODS

KEY RESOURCES TABLE

REAGENT or RESOURCE	SOURCE	IDENTIFIER
**Bacterial and virus strains**		
YYdCas9: BW25993 intC:tetR-dcas9-aadA lacY:ypet-cat	Lawson et al.[3]	N/A
MegaX DH10B T1R Electrocomp™: F- mcrA Δ(mrr-hsdRMS-mcrBC), Φ80lacZΔM15 ΔlacX74 recA1 endA1 araD139 Δ(ara, leu)7697 galU galK λ- rpsL nupG tonA	Invitrogen	Cat# C640003
**Chemicals, peptides, and recombinant proteins**		
Anydrotetracylcine	Roth	Cat#1035708-25MG
Carbenicillin	Roth	Cat#6344.3–25 MG
Ammonium carbonate	Honeywell	Cat#10361-29-2
Isopropanol, Rotisolv ≥99.95%, Ultra LC-MS	Roth	Cat#0733.1
HP-921 and purine in API-TOF Reference Mass Solution Kit	Agilent	G1969-85001
**Deposited data**		
FI-MS data	This paper	https://doi.org/10.5281/zenodo.11563885 and MassIVE: MSV000096254
**Oligonucleotides**		
Oligonucleotides are listed in Table S5	Twist Bioscience	N/A
**Recombinant DNA**		
Ubiquitin WT	Brzovic et al.[19]	Addgene #12647
pgRNA-bacteria	Qi et al.[7]	Addgene #44251
**Software and algorithms**		
MATLAB R2021a	mathworks.com	N/A
FLASHDeconv 2.0 BETA+	https://openms.de/applications/flashdeconv	N/A
MSConvert	https://proteowizard.sourceforge.io	N/A

EXPERIMENTAL MODEL AND STUDY PARTICIPANT DETAILS

Strains and culture

E. coli YYdCas9[3] was the wild-type strain used in this study. All strains in this study derive from the YYdCas9 strain and are listed in the key resources table. MegaX DH10B competent *E. coli* (Cat#C640003, Invitrogen) were used for cloning.

Media

Cultivations were performed with LB medium (Cat#L3522, Sigma-Aldrich) or M9 minimal medium with glucose (5 g/L) as sole carbon source. The M9 medium consists of (per liter): 7.52 g $Na_2HPO_4 \cdot 2\ H_2O$, 5 g KH_2PO_4, 1.5 g $(NH_4)2SO_4$, 0.5 g NaCl. Additionally, the following components were sterile filtered and added separately (per liter M9 medium): 1 mL 0.1 M $CaCl_2$, 1 mL 1 M $MgSO_4$, 0.6 mL 0.1 M $FeCl_3$, 2 mL 1.4 mM thiamine-HCl and 10 mL trace salts solution. The trace salts solution consists of (per liter): 180 mg $ZnSO_4 \cdot 7$ H2O, 120 mg $CuCl_2 \cdot 2\ H_2O$, 120 mg $MnSO_4 \cdot H_2O$, 180 mg $CoCl_2 \cdot 6\ H_2O$. For strains transformed with pgRNA-ubiquitin plasmid or variants, 100 μg/mL carbenicillin (Carb) was added to the media. To induce the expression the dCas9 protein, aTc was supplemented to a final concentration of 200 nM.

METHOD DETAILS

Cloning of pgRNA-ubiquitin

The ubiquitin coding sequence, T7 terminator and RBS were first amplified from the plasmid Ubiquitin WT[19] (Cat#12647, Addgene) and added to pgRNA-bacteria[7] using Golden gate cloning.[20] The promotor J23100 was amplified from a gene fragment that was

ordered from Twist Bioscience (San Francisco, USA) and was added to the pgRNA using IVA cloning,[21] forming pgRNA-ubiquitin. The plasmid was then transformed into *E. coli* YYdCas9 using TSS transformation.[22]

Cloning of CRISPRi strains

sgRNA guide sequences were used from a metabolism-wide CRISPRi library[23] and sequences of ubiquitin barcodes were designed with MATLAB scripts. Single-stranded, 241 bp DNA oligonucleotides were ordered from Twist Bioscience (San Francisco, USA), encoding both a unique ubiquitin barcode and a corresponding sgRNA sequence (Table S5). Oligonucleotides were amplified with PCR. The pgRNA plasmid backbone was linearized by PCR and amplified oligonucleotides were inserted with Gibson assembly.[24] Intermediate cloning was performed in electrocompetent MegaX DH10B *E. coli* (Cat#C640003, Invitrogen). The resulting plasmid pool was purified (Thermo Fisher #K0503) and electroporated into *E. coli* YYdCas9. Single colonies were arrayed into a 96-well format and stored as glycerol stocks. The oligonucleotide inserts were confirmed by sequencing.

Cultivation and sampling of ubiquitin-barcodes and metabolites in shake flasks

For metabolite and ubiquitin-barcode analysis, strains were inoculated from a glycerol stock in 3 mL rich medium (LB) with carbenicillin (100 μg/mL) and cultivated for 8 h at 37°C. Cells were then transferred in 10 mL M9 minimal medium with glucose and carbenicillin for overnight cultivation at 37°C in 100 mL shake flasks. M9-pre-cultures were adjusted to a starting OD_{600} of 0.05 into 10 mL M9 medium. The strains were cultivated for 6.5 h. Subsequently, OD_{600} was measured and an equivalent of 1 OD_{600} was transferred into a 1.5 mL microcentrifuge tube. Cells were then centrifuged for 10 min at 13,000 rpm and 4°C. The supernatant was discarded and cell pellets were resuspended in 1 mL of ice-cold phosphate buffer saline (PBS, Cat#10722497, Invitrogen). Centrifugation and PBS washing was then repeated. The supernatant was discarded and cell pellets were stored at −80°C for 1 h. Finally, pellets were resuspended in 100 μL of 10 mM ammonium carbonate $(NH_4)_2CO_3$ (Cat#10361-29-2, Honeywell), and centrifuged for 10 min at 4°C. The supernatant was stored at −80°C until analysis by FI-MS.

Cultivation and sampling of ubiquitin-barcodes in 96-well plates

Strains were inoculated from a glycerol stock in 1 mL rich medium (LB) with carbenicillin (100 μg/mL) and cultivated in 96-deep-well plates for 7 h at 37°C. Cells were then centrifuged for 10 min at 4,000 rpm and 4°C. The supernatant was discarded and cell pellets were resuspended in 400 μL of ice-cold PBS (Cat#10722497, Invitrogen). The supernatant was discarded and cell pellets were stored at −80°C for at least 1 h. Finally, pellets were resuspended in 100 μL of 10 mM ammonium carbonate $(NH_4)_2CO_3$ (Cat#10361-29-2, Honeywell), and centrifuged for 10 min at 4°C. The supernatant was stored at −80°C until analysis by FI-MS.

Flow-injection mass spectrometry (FI-MS)

For FI-MS, cell extracts were directly injected into an Agilent 6546 Series quadrupole time-of-flight mass spectrometer (Agilent Technologies, USA) as described previously.[9,10] The electrospray source was operated in negative and positive ionization mode. The mobile phase was 60:40 isopropanol:water buffered with 10 mM ammonium carbonate $(NH_4)_2CO_3$ and 0.04% (v/v) ammonium hydroxide for both ionization modes, and the flow rate was 0.15 mL/min. For online mass axis correction, 2-propanol (in the mobile phase) and HP-921 were used for negative mode and purine and HP-921 were used for positive mode. Mass spectra were recorded from 50 to 1700 m/z with a frequency of 1.4 spectra/s for 0.5 min using 10 Ghz resolving power. Source temperature was set to 225°C, with 1 L/min drying gas and a nebulizer pressure of 20 psi. Fragmentor, skimmer, and octupole voltages were set to 120 V, 65 V, and 650 V, respectively. Capillary voltage was set to 3,500 V. For FI-MS of whole living cells, 3 μL of an *E. coli* culture in 1:8 M9-medium:water was injected. The heat-exchanger was set to 90°C, and capillary voltage was 5,000 V.

Raw data files were converted into mzXML files and processed by custom MATLAB scripts. The 32 spectra with the highest signal in the total ion count were summed and baseline adjusted with *msbackadj.m*. Peaks with a minimum peak height of 5000 units and a peak prominence of 5000 units were selected with *findpeaks.m*, and annotated with a 3 mDa tolerance by matching monoisotopic masses of all metabolites in the iML1515 model,[14] considering a single proton loss ([M-H]-) in negative mode and single proton gain ([M + H]+) in positive mode. Positive and negative mode annotation were merged and if a metabolite was annotated in both modes negative mode was selected. For each metabolite, the height of the annotated ion peak was taken for further analysis and normalized to the mean of the control strain to obtain fold-changes. FLASHDeconv[12] was used for spectral deconvolution. Therefore, raw Agilent files (.d files) were converted into mzML format using MSConvert and then processes with FLASHDeconv 2.0 BETA+. The 5 monoisotopic masses determined by FLASHDecon that had the highest summed peak intensity were used for further analysis.

QUANTIFICATION AND STATISTICAL ANALYSIS

Statistical analysis was performed using custom MATLAB scripts. The number of replicates (n) of each experiment can be found in the respective figure caption, and n represents the number of independent shake flask cultures. Significant m/z features were defined with a 4-fold cut-off and a *p*-value<0.01 for a two-sample t-test. Principal component analysis was performed with the FBMN-STATS application (https://fbmn-statsguide.gnps2.org/).

Cell Reports Methods, Volume *4*

Supplemental information

Intact protein barcoding enables one-shot identification of CRISPRi strains and their metabolic state

Vanessa Pahl, Paul Lubrano, Felicia Troßmann, Daniel Petras, and Hannes Link

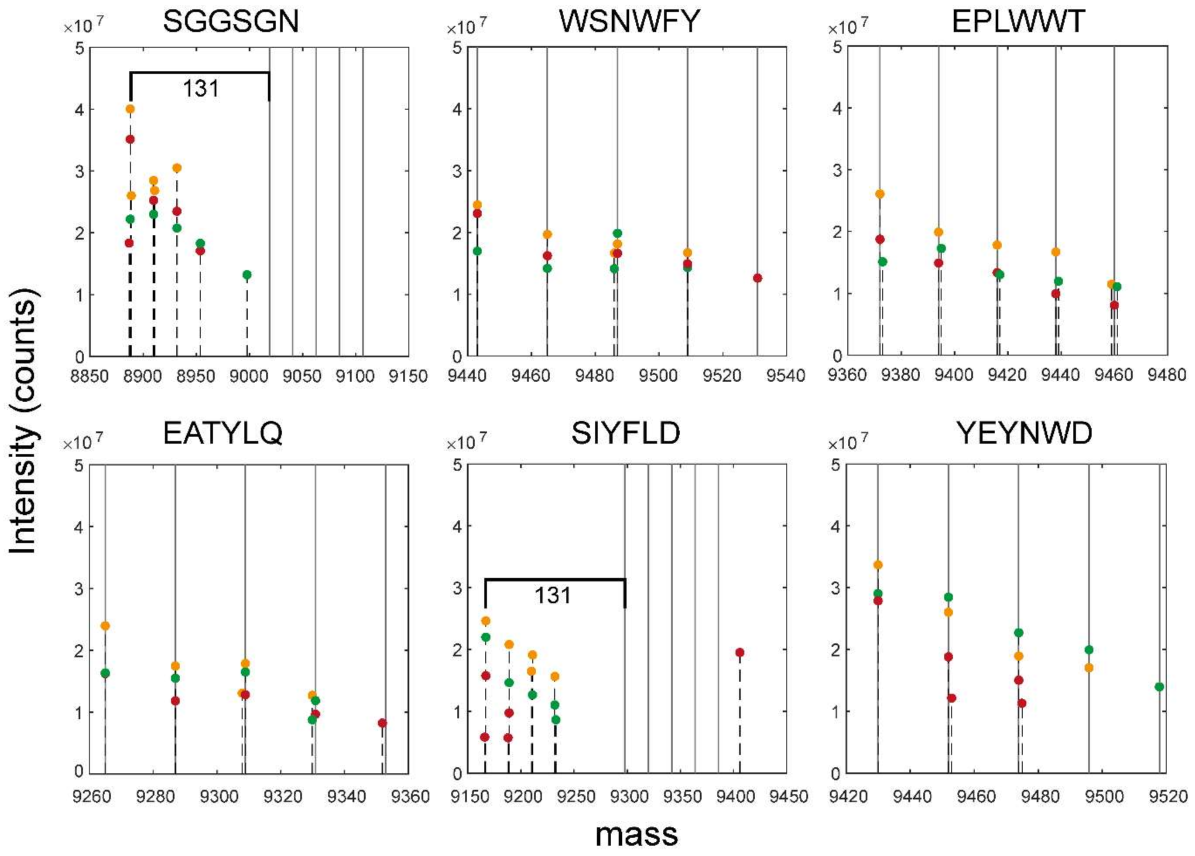

Figure S1. Monoisotopic masses of six ubiquitin barcodes determined by spectral deconvolution of FI-MS data, Related to Figure 3. Shown are the 5 monoisotopic masses with highest summed intensity from n = 3 samples. Dots with different colour and dotted lines are deconvoluted masses of n = 3 replicates. Solid lines are theoretical masses of ubiquitin with the N-terminal barcodes shown above the graph, with 0-4 sodium adducts.

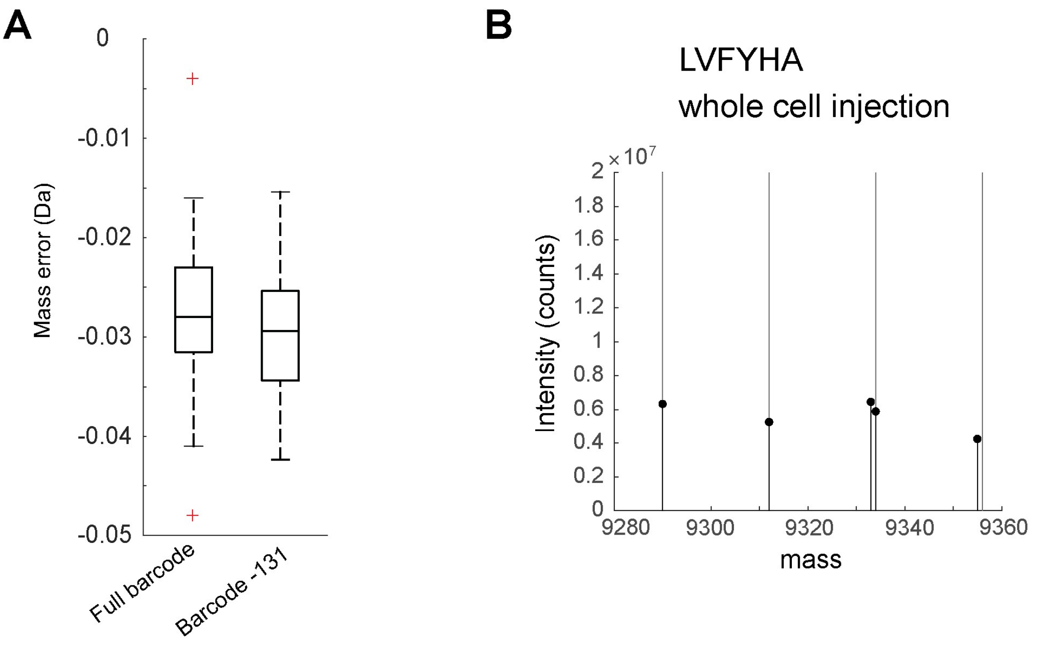

Figure S2. Mass accuracy of protein barcode detection and analysis of the ubiquitin-LVFYHA barcode in whole living cells, Related to Figure 4. A) Mass accuracy of protein barcode detection after spectral deconvolution. Shown is the error for 48 barcodes with correct monoisotopic mass (full barcode), and 38 barcodes with correct monoisotopic mass -131 due to methionine cleavage (barcode -131). **B)** The CRISPRi control strain expressing LVFHA-ubiquitin was diluted in 1:8 M9-medium: water and 3 μL were directly injected into the mass spectrometer. Cells were heated to 90°C in the heat exchanger of the column oven. Shown are 5 deconvoluted masses with highest summed intensities. Dots with dotted lines are deconvoluted masses. Solid lines are theoretical masses of LVFYHA-ubiquitin, with 0-4 sodium adducts.
